# Integrated Infrared Radiation Characteristics of Aircraft Skin and the Exhaust Plume

**DOI:** 10.3390/ma15217726

**Published:** 2022-11-02

**Authors:** Juqi Zhang, Hong Qi, Donghang Jiang, Baohai Gao, Mingjian He, Yatao Ren, Kefu Li

**Affiliations:** 1 School of Energy Science and Engineering, Harbin Institute of Technology, Harbin 150001, China; 2 Key Laboratory of Aerospace Thermophysics, Ministry of Industry and Information Technology, Harbin 150001, China; 3 Faculty of Engineering, University of Nottingham, Nottingham NG7 2RD, UK; 4 Architectural Engineering Institute, North China Institute of Aerospace Engineering, Langfang 065000, China

**Keywords:** infrared radiation, aircraft skin, exhaust plume, Mach number, aerodynamic heating, detection

## Abstract

Infrared radiation (IR) characteristics are important parameters for detecting, identifying, and striking military targets in the context of systematic countermeasures. Accurate calculation of IR characteristics for aircraft is significant for the simulation of war situations and the designation of combat strategy. In this work, integrated IR characteristics of aircraft skin and exhaust plume and their interaction are investigated by considering the reflection based on a bi-directional reflectance distribution function and various influence factors such as solar irradiation, ground reflection, aerodynamic heating, and projection radiation from the background. Combined with infrared emission and reflection characteristics of the skin, omnidirectional IR intensity distributions of 3−5 μm and 8−14 μm at different Mach numbers are obtained. The exhaust plume IR characteristic for different waves and wavebands are also investigated by considering the presence or absence of base and the difference in nozzle inlet temperature. On this basis, integrated IR characteristics between the skin and exhaust plume are investigated. Results show that aircraft IR characteristics of 3−5 μm are concentrated in the exhaust plume and high-temperature skin near the exhaust plume, while the signals of 8−14 μm are concentrated in the skin. The research results are expected to supply guidance for better detection and identification of typical flight targets.

## 1. Introduction

Infrared radiation (IR) characteristics are an important parameter for detecting, identifying, and striking typical military targets such as aircraft in the context of systematic countermeasures [1]. Targets with significant IR characteristics are usually at a disadvantage in the infrared countermeasure system. The study of IR characteristics of typical flight targets is of strategic significance for the simulation of a war situation, the designation of combat strategy, and infrared countermeasures in modern beyond-visual-range operations [2,3].

The measurement of target IR characteristics has received extensive attention in recent years. Infrared characteristics calculation of aircraft is mainly based on the thermodynamics and IR involved in fluid dynamics, materials science, and other multi-disciplinary theories [4,5,6]. Infrared characteristics calculations for aircraft are closely related to skin material properties, aerothermal effects, exhaust plume properties, and environmental conditions. Different characteristics of surface materials, such as emissivity, absorption, and reflectivity, result in different IR properties and, thus, different temperature and radiation distributions [7,8,9,10]. High-speed relative movement of aircraft and the air leads to aerodynamic heating, which is also an important source of radiation [11,12,13]. In addition, due to the high temperature in the core area of the exhaust plume, the IR signal in the visible area near the nozzle will be affected inevitably by the heating effect of IR from the exhaust plume on the skin [14,15,16]. Therefore, it is of great significance to study the individual IR characteristics of aircraft skin and exhaust plume, as well as their interaction.

In the absence of practical experiments, the IR simulations of targets are widely used in the simulation measurement of aircraft radiation characteristics [17,18,19,20,21]. As for IR signal characteristics of aircraft skin and exhaust plume, Lu et al. [22] established the IR signature synthetic model on the basis of the computational fluid dynamics and the reverse Monte Carlo (RMC) method, which can accurately simulate the IR characteristics of an aircraft. Mahulikar et al. [23] proposed a program to predict the infrared signature emissions from the airframe, the engine casing, and the plume and investigated the impact of sun, sky, and earth radiation on an aircraft IR signal. Li et al. [24] proposed a real-time IR characteristic simulation method of aircraft skin based on the panel element method by considering the aerodynamic heating effect, which can help to assess the IR impacts of different environmental factors and materials. Harkiss [25] investigated the effects of bi-directional reflectance distribution functions (BRDFs) incorporated into IR signature simulations based on the radiometric model simulating a large commercial aircraft’s IR intensity of aircraft during take-off and landing states. Taking a solid rocket motor as the research object, Heragu et al. [26] considered the absorption and emission of H_2_O, CO_2_, and Al_2_O_3_ mixture gas, as well as the scattering of Al_2_O_3_ particles, and calculated the infrared signal of exhaust plume by RMC method. However, most of the researchers decomposed the IR characteristics of aircraft into two independent calculation modules: the IR of skin and exhaust plume, which is inaccurate to some extent. Due to the high-temperature exhaust plume in the core area, the IR signal in the visible area near the nozzle will be affected by the heating effect of IR from the exhaust plume on the skin inevitably. In addition, the skin will have a shielding effect on the IR of the exhaust plume.

In the present work, the integrated IR characteristics of aircraft skin and the exhaust plume generated by a convergent-divergent nozzle and their interaction are investigated by considering the BRDF reflection and various influence factors such as solar irradiation, ground reflection, aerodynamic heating, and projection radiation from the background. Corresponding investigations are conducted by the self-programmed C++ code. The remainder of this work is organized as follows. In Section 2, the IR characteristics modeling for aircraft skin is introduced, including the finite element discretization of thermal conductivity differential equation and aerodynamic heating method. Gas radiation characteristics and exhaust plume radiation calculation are presented in Section 3. In Section 4, the results of IR characteristics for aircraft skin and the exhaust plume are exhibited. Section 5 summarizes this work.

## 2. IR Characteristics Modeling for Aircraft Skin

In the three-dimensional Cartesian coordinate system, the differential equation of transient heat conduction is:

(1) $$\rho c_{p}\frac{\partial T}{\partial \tau }=\nabla \cdot \left(k\nabla T\right)+\overset{\cdot }{Q}$$

(2) $$\pi =\Omega_{c}+\Omega_{q}+\Omega_{h}$$

where Ω*_c_*, Ω*_q_*, and Ω*_h_* represent the contribution of heat conduction, external heat flux, and convection, respectively. Solar irradiation, secondary radiation from the ground, atmospheric irradiation, and aerodynamic heating are seen as the external heat flux, and they are gathered into the term Ω*_q_*. The external convective heat transfer is denoted as Ω*_h_*. Ω*_c_*, Ω*_q_*, and Ω*_h_* can be expressed as

(3) $$\Omega_{c}=\frac{1}{2}\underset{V}{∭}\left[k\nabla^{2}T\right]dV$$

(4) $$\Omega_{q}=-\iint_{S_{2}}q^{*}TdS$$

(5) $$\Omega_{h}=\frac{1}{2}\iint_{S_{3}}h{\left(T-T_{\infty }\right)}^{2}dS$$

where $q^{*}$ represents the specified external heat flux in the Neumann boundary condition, in which solar irradiation, secondary radiation from the ground, atmospheric irradiation, and aerodynamic heating are included. *h* is the convective heat transfer coefficient in the Robbin boundary condition and $T_{\infty }$ is the fluid temperature. It is worth noting that Ω*_q_* and Ω*_h_* usually do not occur on the same surface at the same time because the convection effect has been considered in Ω*_q_*. They are introduced into Equation (2) here for derivation and subsequent programming convenience. Therefore, there exist the following comprehensive thermal boundary conditions when the system reaches thermal equilibrium:

(6) $$\begin{array}{l} q_{c}={\left(k\frac{\partial T}{\partial n}\right)}_{n}\\ q^{*}=E_{\mathrm{dir}}+E_{\mathrm{sca}}+E_{\mathrm{ref}}+q_{w}\\ q_{h}=h\left(T-T_{\infty }\right) \end{array}$$

where *q_c_* and *q_h_* are the conduction term and convective term, respectively. *n* is the normal direction of the skin surface. The specific explanation of q^*^ can be seen in Equation (23). The Finite Element Method (FEM), which is programmed by C++ code, is employed to calculate the temperature distribution of the aircraft. Take the three-dimensional tetrahedral element as an example. Assuming that the temperature at four nodes of the tetrahedron is known, the temperature at any position in the unit can be represented as Equation (7)

(7) $$T(P)=N_{i}t_{i}+N_{j}t_{j}+N_{k}t_{k}+N_{m}t_{m}$$

where *T*(*P*) represents the temperature at position *P*. *N* and *t* denote the shape function and temperature of a certain node. Subscript *i*, *j*, *k*, and *m* represent four different nodes. Equation (7) can be rewritten in a matrix form

(8) T=N⋅t

where $N=[N_{i}\;N_{j}\;N_{k}\;N_{m}]$ and $t={\left[t_{i}\;t_{j}\;t_{k}\;t_{m}\right]}^{T}$. For a three-dimensional tetrahedral element, the formula for calculating the shape function is as follows

(9) $$\begin{array}{l} N_{i}=\frac{1}{6V}(a_{i}+b_{i}x+c_{i}y+d_{i}z)\\ N_{j}=\frac{1}{6V}(a_{j}+b_{j}x+c_{j}y+d_{j}z)\\ N_{k}=\frac{1}{6V}(a_{k}+b_{k}x+c_{k}y+d_{k}z)\\ N_{m}=\frac{1}{6V}(a_{m}+b_{m}x+c_{m}y+d_{m}z) \end{array}$$

where *V* is the volume of the tetrahedron. *x*, *y*, and *z* denote the coordinate value of *P* inside the tetrahedron. *a*, *b*, *c*, and *d* are the coefficients related to the coordinate values of nodes, respectively. The expressions of *a_i_*, *b_i_*, *c_i_*, and *d_i_* are as follows

(10) $$\begin{array}{cccc} a_{i}= & \left|\begin{array}{ccc} x_{j} & y_{j} & z_{j}\\ x_{k} & y_{k} & z_{k}\\ x_{m} & y_{m} & z_{m} \end{array}\right| & b_{i}= & -\left|\begin{array}{ccc} 1 & y_{j} & z_{j}\\ 1 & y_{k} & z_{k}\\ 1 & y_{m} & z_{m} \end{array}\right|\\ c_{i}= & -\left|\begin{array}{ccc} x_{j} & 1 & z_{j}\\ x_{k} & 1 & z_{k}\\ x_{m} & 1 & z_{m} \end{array}\right| & d_{i}= & -\left|\begin{array}{ccc} x_{j} & y_{j} & 1\\ x_{k} & y_{k} & 1\\ x_{m} & y_{m} & 1 \end{array}\right| \end{array}$$

where the constants corresponding to the other three nodes of the tetrahedron are calculated based on the alternate circulation of subscripts *m*, *i*, *j*, and *k*. Defining the temperature gradient matrix **g**, the derivative of shape function **B**, and the heat conduction coefficient matrix **D**, Equation (2) can be written as

(11) $$\pi =\frac{1}{2}\underset{V}{∭}\left(g^{T}\mathrm{Dg}\right)dV-\iint_{S_{2}}\left(t^{T}N^{T}q^{*}\right)dS+\frac{1}{2}{\iint_{S_{3}}h\left(t^{T}N^{T}-T_{\infty }\right)}^{2}dS$$

Because of the independence of coordinate and grid temperature, then

(12) $$\frac{\partial \pi }{\partial t}=\underset{V}{∭}\left(B^{T}\mathrm{DB}\right)dVt-\iint_{S_{2}}\left(N^{T}q^{*}\right)dS+\iint_{S_{3}}\left(hN^{T}N\right)dSt-\iint_{S_{3}}\left(N^{T}hT_{\infty }\right)dS$$

Define the stiffness matrix and loading matrix of elements **K**^e^ and **f**^e^ with the following equations

(13) $$K^{e}==\underset{V}{∭}\left(B^{T}\mathrm{DB}\right)dV+\iint_{S_{3}}\left(hN^{T}N\right)dS$$

(14) $$f^{e}=\iint_{S_{2}}\left(N^{T}q^{*}\right)dS+\iint_{S_{3}}\left(N^{T}hT_{\infty }\right)dS$$

The two terms on the right-hand side of Equation (13) represent the contribution of heat conduction and convection, respectively. The two terms on the right-hand side of Equation (14) denote the specified heat flux and the convective heat transfer on the surface, respectively. Generally, the Neumann boundary condition and Robbin boundary condition will not appear on the same surface at the same time for **f**^e^, but they are introduced simultaneously here in order to employ the radiative heat flux into the equation. Substituting Equations (13) and (14) into Equation (12), it can be rewritten into a matrix form:

(15) $$K^{e}\cdot t=f^{e}$$

Equation (15) is the equation systems for a tetrahedron with four grids. Equation systems of the whole region for solving the element temperature values can be obtained by the superposition of stiffness matrix **K**^e^ and loading matrix **f**^e^ of all tetrahedrons. Then, the temperature value of each point in the calculation domain can be obtained.

The boundary conditions, such as external convective heat transfer, solar irradiation, secondary radiation from the ground, atmospheric irradiation, and aerodynamic heating, are added to the finite element matrix through the loading matrix. The radiative heat flux from the sun to the aircraft skin consists of three parts, including solar direct radiation, solar scattering radiation, and secondary radiation from the ground. Solar direct radiation is the main factor affecting the static IR characteristics of the skin. The length of sunlight penetrating the atmosphere is determined by the solar elevation angle and thickness of clouds. In addition, the aircraft skin is also affected by solar scattering radiation, which is caused by gas molecules, dust particles, and water droplets in the atmosphere. The solar direct radiation *E*_dir_ and solar scattering radiation *E*_sca_ of the aircraft skin can be expressed as

(16) $$E_{\mathrm{dir}}=H_{\mathrm{sun}}\cdot p^{m}\cdot \cos\theta$$

(17) $$E_{\mathrm{sca}}=\frac{1}{2}H_{\mathrm{sun}}\sin\tau \frac{1-p^{m}}{1-1.4\ln p}\cos^{2}\frac{\varphi }{2}$$

where *H*_sun_ is the solar constant (1367 W∙m^−2^). *θ*, *𝜏*, and 𝜑 are the solar incident angle, solar elevation angle, and the angle between the plane of the micro-element and the horizontal plane, respectively. *p* is the atmospheric transparency, which is usually related to weather conditions. The values of sunny, cloudy, overcast, and rainy days are 0.85, 0.8, 0.65, and 0.532, respectively. *m* is the atmosphere mass, which is calculated as follows:

(18) $$m=\left\{\begin{array}{cc} \;1/\sin\tau \; & \tau \geq 30{}^{\circ}\\ {\left[1229+{\left(614\sin\tau \right)}^{2}\right]}^{1/2}-614\sin\tau \; & \tau <30{}^{\circ} \end{array}\right.$$

For the aircraft skin, the surface of the exterior normal pointing to the sky is mainly affected by the direct and scattering solar radiation, while the surface with the inclined exterior normal is also affected by the ground reflection. The secondary radiation from the ground *E*_ref_ is equal in all directions of the hemispheric space.

(19) $$E_{\mathrm{ref}}=\rho_{\mathrm{grd}}(E_{\mathrm{dir}}+E_{\mathrm{sca}})$$

where *ρ*_grd_ is the average reflection coefficient of the ground.

Apart from solar radiation, aerodynamic heating is a non-negligible part of the skin heating calculation when aircraft flies at high speed. The fuselage was simplified as a blunt body model, the wing was simplified as a wedge-shaped plate, and the blunt body geometric model was divided into stagnation region, hemispheric surface, and cone surface with the non-stagnation region. To calculate the aerodynamic heating efficiently, researchers proposed a series of engineering predictor methods, such as the Fay−Riddell relation [27] and the Lees formula [28]. Lees formula is widely utilized in the heat flux calculation for stagnation region, which assumes that the heat flux *q*_ws_ at stagnation region is only dependent on the inflow and geometric parameters (see Equation (20))

(20) $$q_{\mathrm{ws}}=2.737\times 10^{-7}{\left(\frac{\gamma_{\infty }-1}{\gamma_{\infty }}\right)}^{0.25}{\left(\frac{\gamma +1}{\gamma -1}\right)}^{0.25}\rho_{\infty }{}^{0.5}u_{\infty }{}^{3}R_{N}{}^{-0.5}$$

where *ρ*_∞_, *u*_∞_, and *γ*_∞_ represent the incoming flow density, incoming flow velocity, and incoming air-specific heat ratio at infinity, respectively; *γ* denotes the specific heat of gas; *R_N_* is the radius of curvature of the wall in a certain point *N*. Heat flux of hemispheric surface for blunt body model can be obtained by the following equation

(21) $$\frac{q_{\mathrm{wb}}}{q_{\mathrm{ws}}}=\frac{2\omega \sin\omega \left[\left(1-\frac{1}{\gamma_{\infty }Ma_{\infty }^{2}}\right)\cos^{2}\omega +\frac{1}{\gamma_{\infty }Ma_{\infty }^{2}}\right]}{\left(1-\frac{1}{\gamma_{\infty }Ma_{\infty }^{2}}\right)\left(\omega^{2}-\frac{\omega \sin4\omega }{2}+\frac{1-\cos4\omega }{8}\right)+\frac{4}{\gamma_{\infty }Ma_{\infty }^{2}}\left(\omega^{2}-\omega \sin2\omega +\frac{1-\cos2\omega }{2}\right)}$$

where *Ma*_∞_ is the Mach number at infinity. *ω* is the central angle based on the body axis. For the blunt cone body with a non-stagnation region, the heat flux *q*_wc_ can be obtained by solving the Stanton number of the flow around the cone:

(22) qwc=ρeuecp0.575Rexρ∗u∗ρeue1/2Pr−3/2Te+Pr1/2(T0−Te)−Tw

where *ρ*_e_ and *u*_e_ represent density and velocity for the outer boundary layer, respectively. *T*_0_, *T*_e_, and *T*_w_ are the initial temperature and the outer edge temperature of the boundary layer and wall, respectively. ρ^*^ and u^*^ represent the density and velocity in the reference enthalpy state. Re and Pr are the Reynolds number and Prandtl number, respectively. It is worth noting that the specified heat flux q^*^ in the loading matrix of element **f**^e^ consists of the solar direct radiation *E*_dir_ and solar scattering radiation *E*_sca_

(23) $$q^{*}=E_{\mathrm{dir}}+E_{\mathrm{sca}}+E_{\mathrm{ref}}+q_{w}$$

where *q*_w_ denotes the heat flux of aerodynamic heating *q*_ws_, *q*_wb_, or *q*_wc_, the selection of which depends on the corresponding boundary condition of the element.

**Figure 1:**
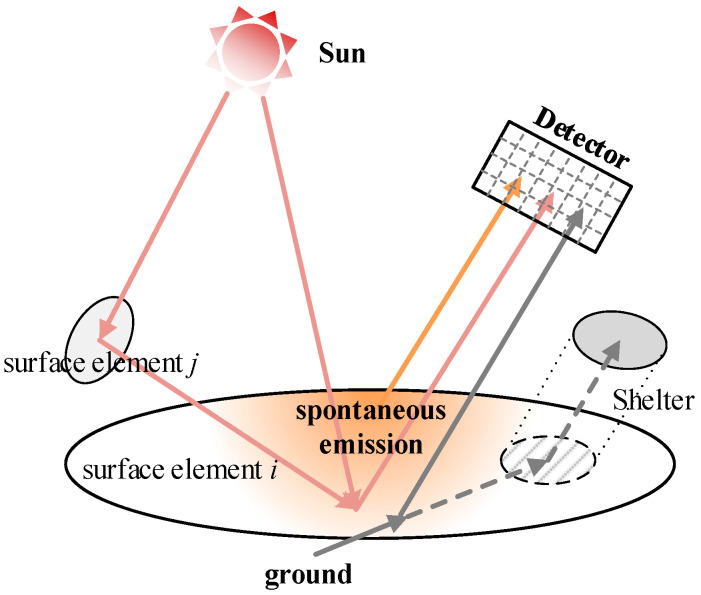
Schematic diagram of skin infrared radiation intensity calculation.

After obtaining aircraft-skin temperature based on the FEM and boundary condition for solving temperature, the IR intensity of skin at a specific detection angle can be calculated. As seen in Figure 1, the IR intensity calculation mainly consists of three parts, i.e., long wavelength emission due to the existence of skin temperature, medium wavelength reflection from the sun, and long wavelength reflection from the ground. In this work, two atmospheric windows (mid-infrared waveband 3−5 μm and far infrared waveband 8−14 μm) are investigated. Dividing the detection band into *N* spectral bands, the hemispherical radiation of any triangular surface element *i* on the aircraft skin can be calculated by the following equation

(24) $$\begin{array}{l} A_{i}E_{i}=\sigma \sum_{n=1}^{N}A_{i}\varepsilon_{i}^{n}B_{T_{i}}^{n}T_{i}^{4}+H_{\mathrm{sun}}\sum_{n=1}^{N}\left(1-\alpha_{i}^{n}\right)B_{T_{\mathrm{sun}}}^{n}\left(A_{i,\mathrm{pro}}^{\mathrm{sun}}P_{\mathrm{irr}}+A_{i}P_{\mathrm{sca}}\right)\\ \;\;\;+H_{\mathrm{sun}}\sum_{n=1}^{N}\sum_{j=1}^{M}X_{j,i}^{}\left(1-\alpha_{i}^{n}\right)\left(1-\alpha_{j}^{n}\right)B_{T_{\mathrm{sun}}}^{n}\left(A_{j,\mathrm{pro}}^{\mathrm{sun}}P_{\mathrm{irr}}+A_{j}P_{\mathrm{sca}}\right)\\ \;\;\;+H_{\mathrm{grd}}\sum_{n=1}^{N}\left(1-\alpha_{i}^{n}\right)B_{T_{\mathrm{grd}}}^{n}A_{i,\mathrm{pro}}^{\mathrm{grd}} \end{array}$$

where $A_{i}$, $A_{i,\mathrm{pro}}^{\mathrm{sun}}$, and $A_{i,\mathrm{pro}}^{\mathrm{grd}}$ denote the area of surface element *i*, the projected area of surface element *i* relative to solar irradiation, and the projected area of surface element *i* relative to ground irradiation, respectively. $A_{j}$ and $A_{j,\mathrm{pro}}^{\mathrm{sun}}$ denote the area of surface element *j* and the projected area of surface element *j* relative to solar irradiation, respectively. *H*_grd_ is the heat flux emitted from the ground, respectively. $B_{T_{i}}^{n}$, $B_{T_{\mathrm{sun}}}^{n}$, and $B_{T_{\mathrm{grd}}}^{n}$ represents the blackbody radiation energy share corresponding to the temperature of surface element *i*, the sun, and the ground in the *n*-th spectral band. $\varepsilon_{i}^{n}$ and $\alpha_{i}^{n}$ are the emissivity and absorptivity of surface element *i* in the *n*-th spectral band, respectively. *M* is the amount of surface element *j* that reflects solar radiative energy to surface element *i*. *X_j_*_, *i*_ is the angle coefficient of surface element *i* and surface element *j*. It is worth noting that the first term on the right-hand side of Equation (24) indicate the skin’s self-emission, which depends on the skin’s temperature and emissivity.

However, the infrared signal of surface element *i* may not be received by the detector placed with a certain angle, so the radiation intensity of surface element *i* received by the detector in a certain waveband is

(25) $$E_{i}^{\det}=\frac{E_{i}}{\pi }\cos\theta_{i}\cdot R_{i}^{\det}$$

where *θ_i_* is the angle of detector normal and plane element normal. $R_{i}^{\det}$ is the shielding factor of surface element *i* for this detection angle, which is defined as 0 when the surface element *i* is shielded and 1 when it is not shielded.

## 3. Gas Radiation Characteristics and Exhaust Plume Radiation Calculation

### 3.1. Line-by-Line Calculation

In the last decades, much attention has been paid to the development of gas radiation characteristics calculation models. The most accurate prediction method of the gas spectral radiative properties is the so-called line-by-line calculation (LBL), which calculates the spectral absorption coefficient for the specified spectral location based on spectroscopic databases containing a set of spectral line parameters [29]. The database utilized in this work is HITRAN2020 [30]. The spectral absorption coefficient at a central wavenumber *η* can be expressed as:

(26) $$\kappa_{\eta }=\sum_{i}\kappa_{\eta }^{i}=\sum_{i}S_{i}F(\eta -\eta_{0i})$$

where $\kappa_{\eta }^{i}$ is the absorption coefficient contribution of the *i*-th spectral line at *η* within the truncated wavenumber (set as 10 cm^−1^ here); *S_i_* and *η*_0*i*_ are the integrated line intensity and central wave number of the *i*-th spectral line. The detailed expression of line intensity *S_i_* can be obtained in Ref. [31]. In the research of the exhaust plume with medium temperature and 1 atm pressure where collision broadening is dominant, a spectral line follows the Lorentz profile expressed as

(27) $$F(\eta -\eta_{0i})=\frac{1}{\pi }\frac{r_{L}}{{\left(\eta -\eta_{0i}\right)}^{2}+r_{L}^{2}}$$

where *r_L_* is half width, defined as

(28) $$r_{L}={\left(\frac{T_{0}}{T}\right)}^{n_{\mathrm{air}}}\left[r_{\mathrm{air}}\frac{P-P_{a}}{P_{0}}+r_{\mathrm{self}}\frac{P_{a}}{P}\right]$$

where *T*, *n*_air_, *P*, *P_a_*, *r*_air_, *r*_self_ are the gas temperature, coefficient of temperature dependence, the total pressure, the gas partial pressure, the air-broadened half-width, and the self-broadened half-width, respectively. *P*_0_ and *T*_0_ are the pressure and temperature under standard conditions, respectively.

### 3.2. Statistical Narrow Band Model

Although LBL has the highest accuracy in the spectral properties calculation of gas, it cannot meet the real-time calculation requirements for the IR characteristics. The statistical narrow band model (SNB) has a trade-off between accuracy and computational time, including the standard, Goody [32], and Malkmus SNB [33]. Among all the narrow band models, the Malkmus SNB has the highest computational accuracy, which provides the narrow band averaged transmissivity given as

(29) $$\bar{\tau_{\eta }}(x)=\exp\left\{-2\frac{\bar{\gamma }}{\bar{d}}\left[{\left(1+xPl_{m}\bar{\kappa }\frac{\bar{d}}{\bar{\gamma }}\right)}^{\frac{1}{2}}-1\right]\right\}$$

where $\bar{\gamma }$, $\bar{d}$, and $\bar{\kappa }$ are the average half-width, spacing, and absorption coefficient of the spectral line, respectively. *x*, *P*, and *l_m_* are the molar fraction, total pressure, and path length, respectively. $\bar{\kappa }$ can be obtained by averaging the intensity *S_i_* of *N* lines within the Δ*η* interval:


(30) $$\bar{k}=\frac{1}{\Delta \eta }\sum_{i=1}^{N}S_{i}$$

$\bar{d}$ can be obtained by the following equation

(31) $$\bar{d}=\frac{\bar{\kappa }\cdot \bar{\gamma }}{{\left(\frac{1}{\Delta \eta }\sum_{i=1}^{N}\sqrt{S_{i}}\right)}^{2}}$$

In addition, $\bar{\gamma }$ is often calculated by empirical formulas based on the different gas components. In this work, the LBL is employed to calculate the gas characteristic for a single wavelength. Considering the computational accuracy and efficiency comprehensively, the SNB is employed to calculate the gas characteristic for the waveband.

### 3.3. Line-of-Sight Method

Under the condition of perfect combustion of an aero-engine exhaust plume, a participating medium can be viewed as pure absorption gas. With the gas characteristic calculated by LBL or SNB, the spectral radiation intensity for pure absorption medium can be solved efficiently by using the line-of-sight (LOS) method [34,35]. By ignoring the scattering effect, the radiation transfer equation is

(32) $$\frac{dI_{\lambda }(s,s)}{ds}=\kappa_{\lambda }(s)I_{b\lambda }(s)-\kappa_{\lambda }(s)I_{\lambda }(s,s)$$

where $\kappa_{\lambda }(s)$, $I_{b\lambda }(s)$, and $I_{\lambda }(s,s)$ are the absorption coefficient of the gas, spectral radiation intensity of blackbody, and spectral radiation intensity along the **s** direction at position *s*, respectively. Considering the formula *τ_λ_*(*s*) = *κ_λ_*(*s*)d*s*, the above equation can be rewritten as the differentiation with respect to the spectral optical thickness

(33) $$\frac{dI_{\lambda }(\tau_{\lambda },s)}{d\tau_{\lambda }}=I_{b\lambda }(s)-I_{\lambda }(\tau_{\lambda },s)$$

Therefore, the spectral radiation intensity generated by the *i*-th grid in the **s** direction can be expressed as

(34) $$I_{\lambda ,i}(s)=I_{\lambda ,i-1}(s)\exp\left(-\tau_{\lambda ,i}\right)+I_{b\lambda ,i}\left[1-\exp\left(-\tau_{\lambda ,i}\right)\right]$$

Integrating Equation (34) along the opposite direction of the detection ray, the boundary emission spectral radiation intensity along the **s** direction can be obtained by

(35) $$I_{\lambda }\left(s\right)=I_{b\lambda ,n}\left[1-\exp^{-\tau_{\lambda .n}}\right]+\sum_{i=1}^{n-1}\left[\exp\left(-\sum_{j=i+1}^{n}\tau_{\lambda .j}\right)-\exp\left(-\sum_{j=i}^{n}\tau_{\lambda .j}\right)\right]I_{b\lambda ,i}$$

where *n* is the number of grids that the ray intersects. $\tau_{\lambda ,j}$ is the spectral optical thickness of the *j*-th grid and $\tau_{\lambda ,j}=\kappa_{\lambda ,j}ds_{j}$. $I_{b\lambda ,i}$ denotes the blackbody spectral radiation intensity of the *i*-th grid with temperature *T_i_*, which can be obtained by Planck’s law.

## 4. Results and Discussion

### 4.1. Aircraft-Skin IR Characteristics Analysis

**Figure 2:**
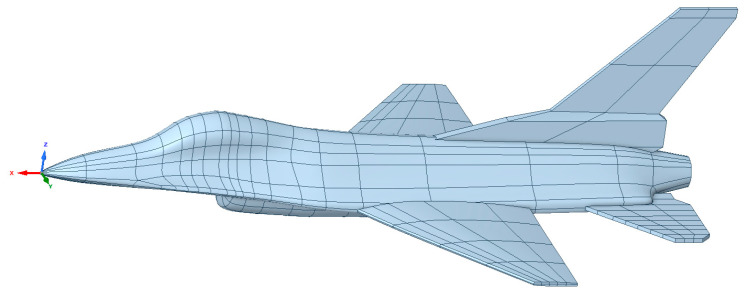
Geometric model of a certain type of single-engine fighter.

In this work, a certain type of single-engine fighter is considered, whose IR characteristics calculation is divided into two parts: skin and exhaust plume. We moderately simplify the complex surface of the model and retain its main features. The aerodynamic heating of friction between skin and air, the heating by the sun, and the projection radiation from the background are considered. All the research is investigated in a steady state. The simplified geometric model of a single-engine fighter is shown in Figure 2. The model has a length of 15.19 m, a width of 9.86 m, and a height of 4.85 m. It is assumed that there is no yaw angle, roll angle, and pitch angle. Take the west direction of the earth's surface where the target is located as the positive direction of the x-axis of the coordinate system, the south direction as the positive direction of the y-axis, and the outer normal direction as the positive direction of the z-axis.

**Figure 3:**
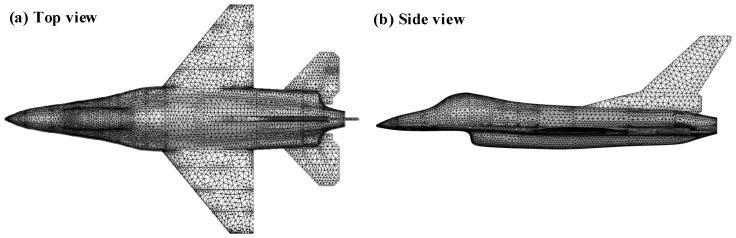
(**a**) Top view and (**b**) side view of grid division for the aircraft.

The finite element analysis software Gid (official version 15.0) is utilized to obtain the unstructured meshing. To save the bandwidth occupied by the finite element calculation of the computer memory resources, the meshes of the nose, cockpit, and tail nozzle parts with large changes in the surface curvature of the model are refined, and the mesh size of the remaining parts is set to 0.2, which can be seen in Figure 3.

**Figure 4:**
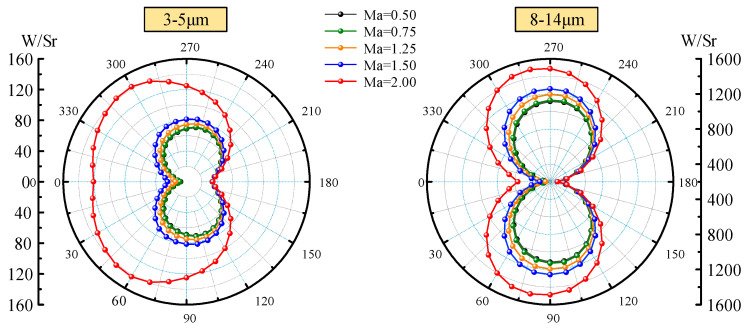
Integral radiation intensity distribution in XOY plane.

Effect of Mach number (0.5, 0.75, 1.25, 1.5, and 2.0) on IR characteristics of aircraft skin is investigated. The self-emission of aircraft skin and the BRDF reflection are comprehensively considered. The calculation time and latitude are set to 12 o ‘clock on March 21 and 0°. A detection point is taken every 10° in the XOY plane and XOZ plane, respectively. The absorptivity and emissivity of the skin surface are set as 0.8, the inlet temperature and ground temperature are both set as 278.15 K, and the emissivity and reflectance of the ground are set as 0.6 and 0.15, respectively. The different Mach number lead to different aerodynamic heating. According to Equations (18)–(20), Mach number affects the heat flux of the stagnation region, hemispheric surface, and cone surface with the non-stagnation region, and thus affects the radiation intensity distribution of aircraft. Figure 4 shows the circumferential integral radiation intensity distribution of the waveband 3−5 μm and the 8−14 μm in the XOY plane when the attack angle is 0°. Since there are no yaw and roll angles of aircraft, the radiation intensities of the two wavebands are distributed symmetrically. The radiation intensity in both wavebands increases with the Mach number. We can see that the radiation intensity of the 8−14 μm waveband is much higher than that of the 3−5 μm waveband, and its peak value is more than 10 times that of the 3−5 μm waveband. For the 3−5 μm waveband, the radiation intensity increases dramatically when the Mach number is more than 1.5, which is because of the sharp increase in the temperature of the head and the inlet. For the 8−14 μm waveband, with the increase in Mach number, the increase in radiation intensity is relatively uniform. Since the emission in this waveband mainly comes from low-temperature emission, the larger the angle of the viewable area, the larger the value of radiation intensity.

**Figure 5:**
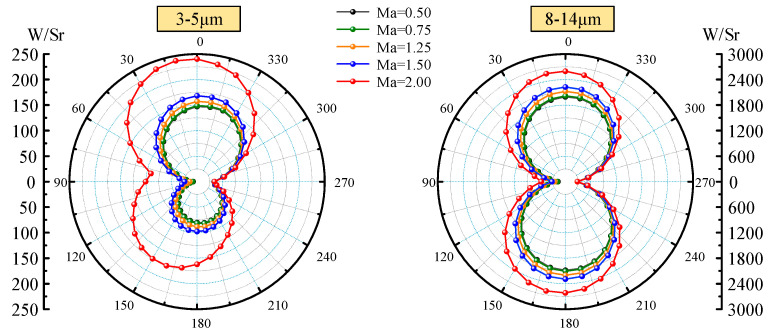
Integral radiation intensity distribution in XOZ plane.

Figure 5 shows the integral radiation intensity distribution in the XOZ plane. For the 3−5 μm waveband, the radiation intensity value of the upper surface of the skin is slightly higher than that of the lower surface of the skin because of the existence of solar irradiation on the upper surface in the 3−5 μm waveband. In addition, the position of the maximum radiation intensity for the upper surface always appears near 0°. The position of the maximum radiation intensity for the lower surface gradually changes from 180° to 150° as the Mach number increases, which is because of the aerodynamic heating of the lower surface intake port. For the 8−14 μm waveband, the radiant intensity values of the upper and lower surfaces are almost equal, and the values of the lower surface are slightly larger. This is because the radiation of the skin material in the 8−14 μm waveband is very large, which can counteract the secondary radiation from the ground.

### 4.2. Exhaust Plume IR Characteristics Analysis

Besides the skin, exhaust plume IR characteristics are also an important part of aircraft IR characteristics analysis. The convergent-divergent nozzle is one of the most commonly used nozzle types for modern aircraft. Generally, when the afterburner is not opened, the inlet temperature of the nozzle is about 800 K, while it can reach more than 1000 K when the afterburner is opened. Therefore, in this sub-section, we investigated the IR characteristics of the Exhaust plume.

**Figure 6:**
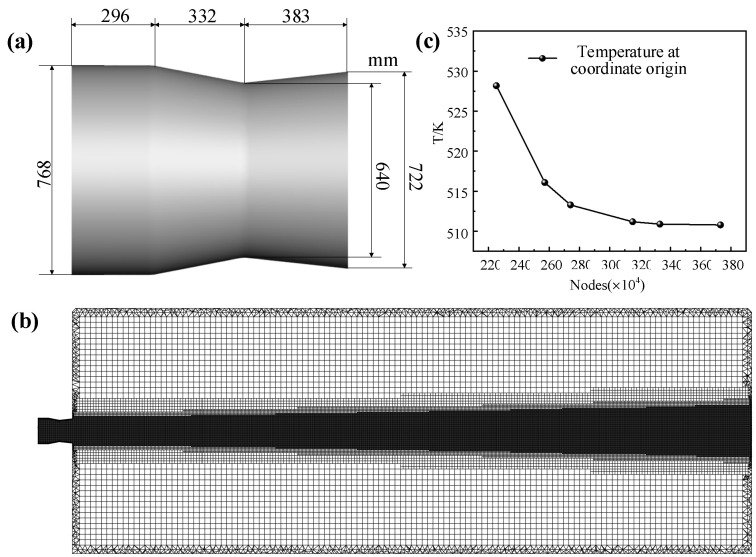
(**a**) Geometric model of Laval nozzle, (**b**) the grid partition of its exhaust plume, and (**c**) grid independence verification for exhaust plume.

Figure 6a,b illustrate the geometric model of the Laval nozzle and the grid partition of its exhaust plume. The inlet, throat, and outlet diameters are 768 mm, 640 mm, and 722 mm, respectively. The length of the inlet, shrinking, and expanding segments are 296 mm, 332 mm, and 383 mm, respectively. Considering that the length of the tail jet usually exceeds the length of the aircraft, the diameter of the flow field downstream of the nozzle is set to 7220 mm, and the length of the flow field is 20,000 mm. The boundary conditions of the nozzle inlet are set as pressure inlet, and the nozzle wall is a wall boundary condition. The side and end face of the cylindrical exhaust plume flow field downstream of the nozzle are both set as pressure outlets. Two-equation k-omega SST turbulence model is employed. The component transport is considered as carbon-monoxide-air, in which the mass fractions of O_2_, CO_2_, CO, and H_2_O at the nozzle inlet are 0.252, 0.0561, 0.00025, and 0.04, respectively, and there is no other component. The mass fraction of O_2_ at each pressure outlet is set as 0.246. In addition, the steady-state model is utilized for calculation. The grid independence verification for the exhaust plume was carried out (see Figure 6c), which indicates that grid number 3326171 has the best trade-off between efficiency and accuracy. For the exhaust plume calculation, the nozzle inlet temperature and the ambient temperature are set as 705 K and 300 K, respectively.

**Figure 7:**
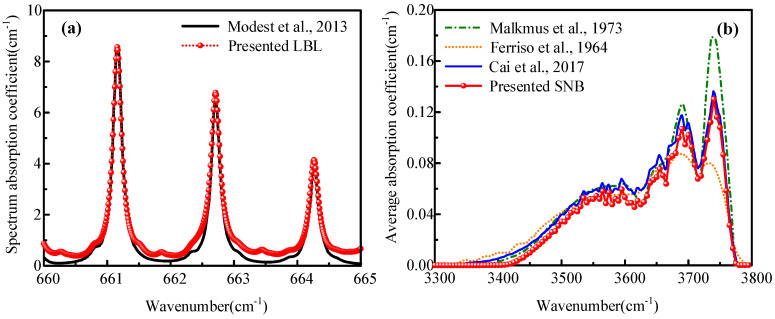
Verification of (**a**) LBL and (**b**) SNB model compared with Refs. [36,37,38,39].

In this sub-section, the LBL is utilized to calculate the spectral absorption coefficients of CO_2_, H_2_O, and CO in the exhaust plume. The SNB is employed to calculate the gas IR characteristics of the waveband. To verify the correctness of LBL and SNB self-programmed by C++ code in the present work, the results are compared with those of Modest [36] and Cai et al. [37,38,39], respectively, as shown in Figure 7. The version of the database used in this work and used in references are HITRAN2020 and HITRAN2008, respectively. We can see that on the three absorption peaks for Figure 7a, the calculated results of LBL in this work are in good agreement with the reference. The slight differences in four weak absorption regions are due to the large number of molecular weak lines incorporated in HITRAN2020 compared to the earlier database. The results of SNB are also consistent with the references.

**Figure 8:**
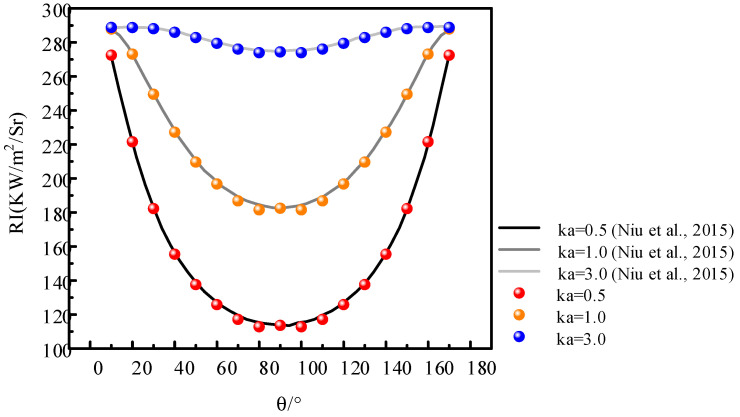
Verification of LOS method compared with Ref. [40].

In addition, the results obtained by self-programmed LOS are compared with those of Ref. [40] (see Figure 8). The cylinder with a radius of 0.5 m and a length of 20 m is employed. The medium temperature is set as 2000 K, and the absorption coefficients are 0.5 m^−1^, 1.0 m^−1^, and 3.0 m^−1^, respectively. The angle between the detection ray and the cylindrical axis increases from 10° to 170°. We can see that the results are in good agreement with Ref. [40], which proves the correctness of the LOS programmed in the present work.

The lateral and backward spectral radiation intensities of convergent-divergent nozzle are investigated at two wavelengths of 2.75 μm and 4.35 μm. It can be seen that the peaks of lateral and backward spectral radiation intensity at 4.35 μm are 160 and 34 W∙m^−2^∙Sr^−1^∙μm^−1^, respectively, which are much greater than those at the 2.75 μm wavelength. At 4.35 μm, the infrared characteristics of the radial expansion of the core region of the exhaust plume can be well captured, while only the high-temperature region near the nozzle diameter can be captured at 2.75 μm. This is because of the larger blackbody radiation intensity at 4.35 μm. In addition, the medium has a larger spectral absorption coefficient at 4.35 μm than 2.75 μm, which indicates the stronger emission effect of exhaust plume at this wavelength.

**Figure 9:**
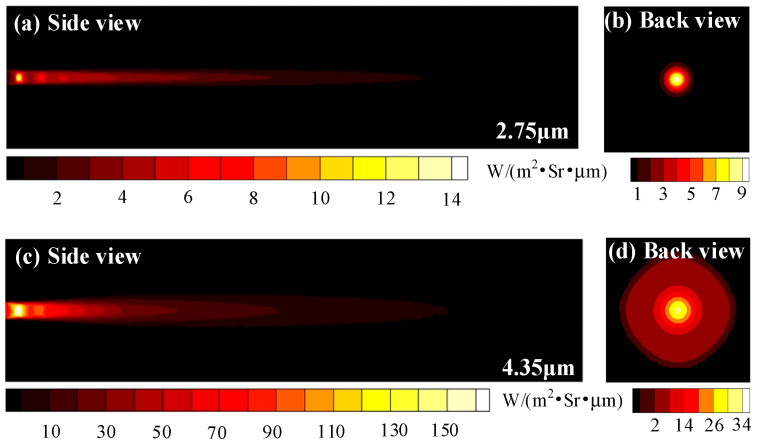
Lateral and backward spectral radiation intensity distributions of exhaust plume at (**a**,**b**) 2.75 μm and (**c**,**d**) 4.35 μm.

**Figure 10:**
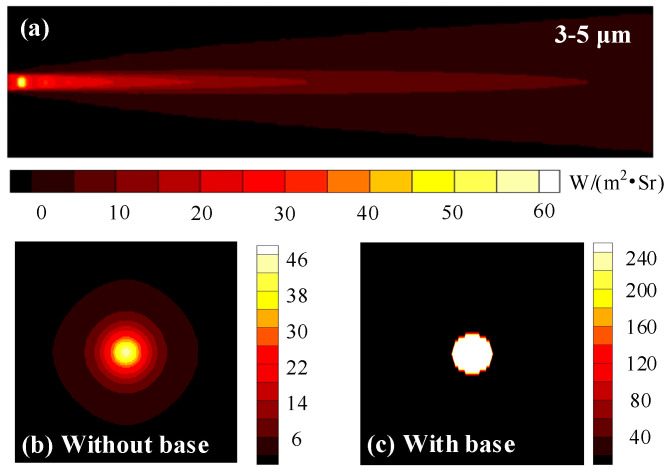
Lateral and backward spectral radiation intensity distributions of exhaust plume in the 3−5 μm waveband. (**a**) Lateral spectral radiation intensity distribution, (**b**,**c**) backward spectral radiation intensity distributions without and with the base.

Then, we investigate the lateral and backward spectral radiation intensity distributions in the 3−5 μm waveband, which are the integration of the radiation intensity of all narrow wavebands in the 3−5 μm waveband based on the single wavelength distribution in Figure 9. We can see from Figure 10 that the radiation intensities gradually decay along the axial and radial directions. In the high-temperature region of the Mach ring at the nozzle outlet, the radiation intensity has a maximum value of 59.7793 W∙m^−2^∙Sr^−1^. Due to the effect of the Laval nozzle, the exhaust plume emerges from the nozzle outlet with a large axial velocity and almost no thermal diffusion in the radial direction. The expansion of the high-temperature airflow in the rear section of the exhaust plume leads to an increase in the radial thickness and a gradual attenuation of the temperature of the exhaust plume, both of which cause a decrease in radiation intensity. Simplifying the visible high-temperature component of the nozzle as a circular base that has the same diameter as the nozzle, backward spectral radiation intensity distributions of exhaust plumes with and without a base are investigated. It can be seen in Figure 10b,c, the existence of a high-temperature wall makes the IR signal in the backward direction much larger than that in the lateral direction.

**Figure 11:**
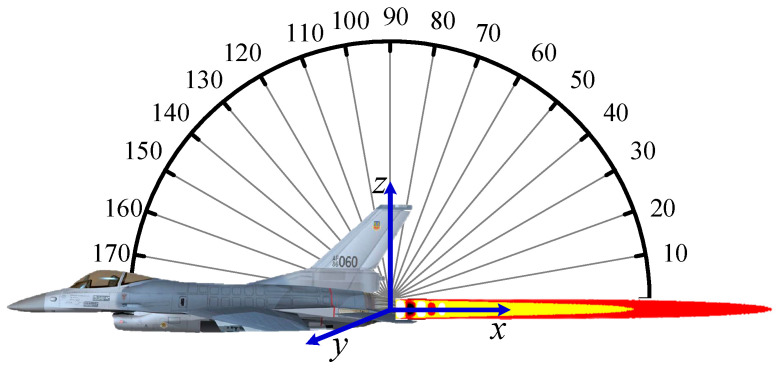
Illustration of detection angle for exhaust plume radiation.

Integrating Figure 10 on the area of the detection field of view and setting different detection angles, as shown in Figure 11, we can obtain the integral radiation intensity distribution in the 3−5 μm waveband under different zenith and circumferential angles. The resolution of the narrow-band wavenumber is 25 cm^−1^, and the detection angle is taken every 10°.

**Figure 12:**
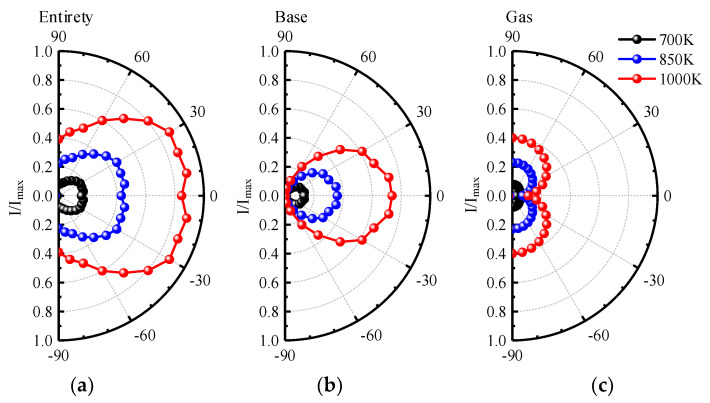
Normalized integral radiation intensity distribution of (**a**) entirety, (**b**) base, and (**c**) gas of 3−5 μm waveband for different nozzle inlet temperatures in XOZ plane.

Figure 12 shows the normalized integral radiation intensity distribution for different nozzle inlet temperatures in the XOZ plane. The shielding effect of the aircraft skin on the gas radiation is not considered. We can see that the maximum radiation intensity appears at the upper and lower sides of the exhaust plume. For the integral radiation intensity distribution of entirety, when the nozzle inlet temperature is 1000 K, the maximum radiation intensity is 5.1 times the corresponding maximum value when the nozzle inlet temperature is 700 K. Therefore, in the 3−5 μm waveband, the aircraft with afterburner is an important factor affecting the gas radiation. With the increase in nozzle inlet temperature, the radiation intensity increases exponentially. In addition, at the same temperature level, the contribution of the integral radiation intensity distribution of the base is much greater than that of gas.

### 4.3. Integrated IR Calculation of Skin and Exhaust Plume

Due to the high temperature in the core area of the exhaust plume, the IR signal in the visible area near the nozzle will be affected inevitably by the heating effect from the exhaust plume on the skin. In addition, the skin will have a shielding effect on the IR of the exhaust plume. Therefore, in this sub-section, integrated calculation of skin and exhaust plume IR is investigated based on the research studies mentioned above.

**Figure 13:**
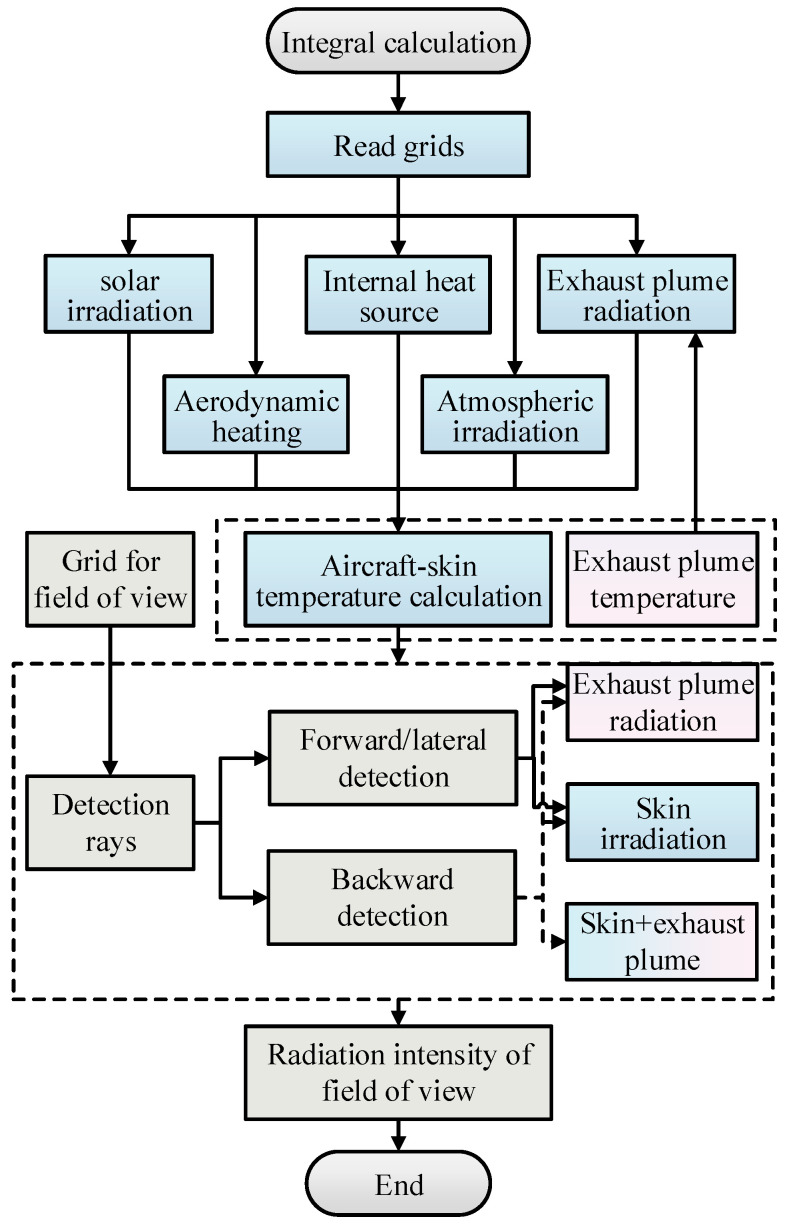
Flowchart of the integrated calculation of skin and exhaust plume IR.

Figure 13 exhibits the flowchart of the integrated calculation of skin and exhaust plume IR. For the skin, the finite element method is used to solve the skin temperature distribution. Apart from the boundary conditions described in Section 2, the boundary condition of the heating effect of the IR from the exhaust plume on the back of the nozzle is added. For the exhaust plume, the Malkmus SNB model is employed to calculate the spectral absorption coefficient of exhaust plume gas, and LOS is utilized to calculate the radiation intensity of the exhaust plume. For infrared detection, “detection rays” are emitted from the grid center of the detection plane based on the idea of the RMC method. The shielding effect of the skin and the attenuation effect of the exhaust plume are considered comprehensively. If the detection ray only intersects with the skin surface, only the IR from the skin is considered in this detection grid. If the detection ray passes through the exhaust plume region and does not intersect with the skin surface, only the IR from the exhaust plume is considered in this detection grid. In addition, if the detection ray passes through the exhaust plume and finally falls on the skin surface, the surface radiation of this point is regarded as the incident radiation intensity in LOS calculation, and the integrated IR signal of the skin and exhaust plume under this detection grid is calculated.

**Figure 14:**
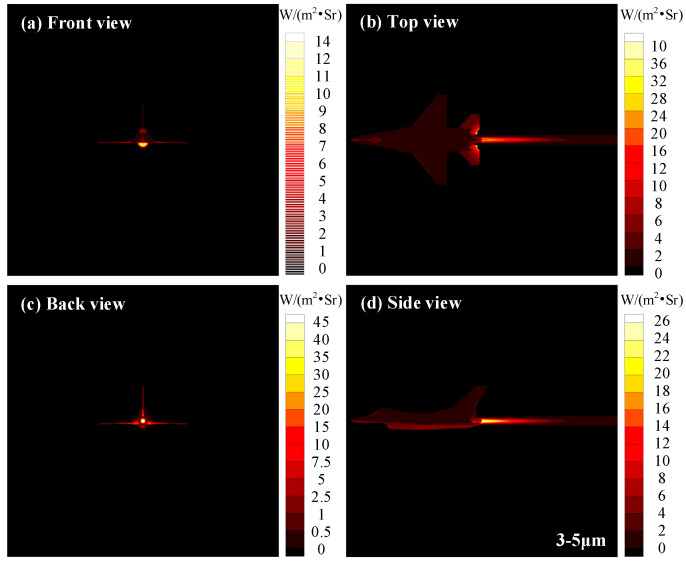
Infrared images of aircraft in 3−5 μm waveband considering radiative heating effect of exhaust plume.

Figure 14 exhibits the infrared images of aircraft in a 3−5 μm waveband considering the radiative heating effect of the exhaust plume. The radiation intensity distribution of the front view shows that the maximum IR is located near the inlet lip, which is due to the complex aerodynamic shape in this area and, thus, the great influence of aerodynamic heating. From the back view, the nozzle region corresponds to the highest radiation intensity of 45 W∙m^−2^∙Sr^−1^, which is due to the radiation intensity superposition of the high-temperature skin and the core region of the exhaust plume under the back view. As can be seen from the top and side views, the maximum radiation intensity is not in the core area of the exhaust plume but in the skin of the nozzle and the horizontal rear wing near the nozzle because of the high temperature of these two locations, and there is neither other skin shielding nor absorption attenuation of the exhaust plume gas.

**Figure 15:**
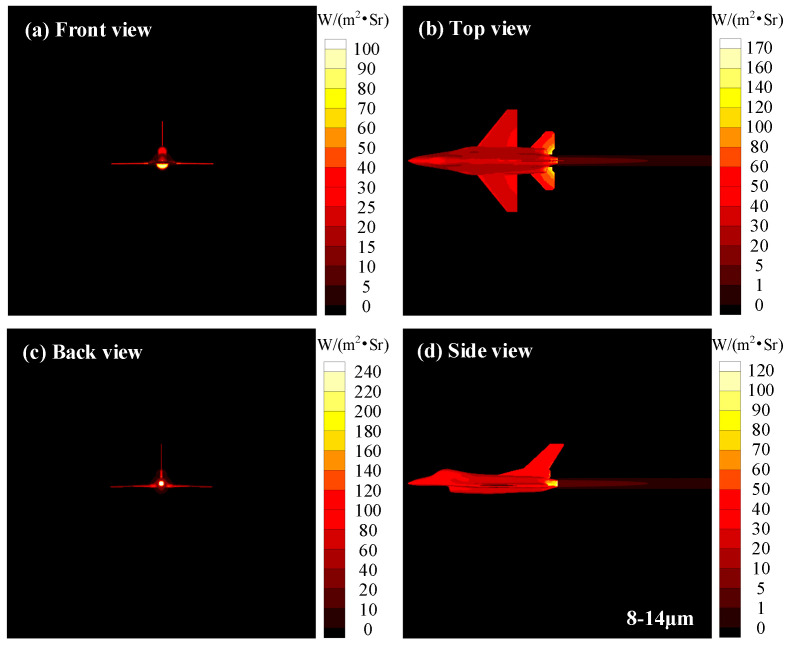
Infrared images of aircraft in 8−14 μm waveband considering radiative heating effect of exhaust plume.

Infrared images of aircraft in 8−14 μm waveband considering the radiative heating effect of exhaust plume are presented in Figure 15. Different from the results for the 3−5 μm waveband, the IR of aircraft skin is much larger than that of exhaust plume in the 8−14 μm waveband. In addition, the aircraft IR signals of 8−14 μm waveband are concentrated in the skin, while the IR characteristic distributions of 3−5 μm waveband are concentrated in the exhaust plume and the high-temperature skin near the exhaust plume. overall, detectors operating in the 8−14 μm waveband are more likely to capture most of the skin IR signals, while the detectors working in the 3−5 μm waveband are more likely to capture the IR signals of the exhaust plume and the visible high-temperature parts of the nozzle.

Infrared radiation (IR) characteristics are important parameters for detecting, identifying, and striking military targets in the context of systematic countermeasures. Targets with significant IR characteristics are usually at a disadvantage in infrared countermeasure systems. To decrease the IR signal of aircraft skin and nozzle significantly, it is necessary to reduce the visible area and adopt the low-emissivity and low-reflectance skin materials, according to the research investigated above.

## 5. Conclusions

In the present work, the integrated IR characteristics of aircraft skin and the exhaust plume generated by a convergent-divergent nozzle, and their interactions are investigated by considering the BRDF reflection and various influence factors such as solar irradiation, ground reflection, aerodynamic heating, and projection radiation from the background. Combined with the infrared emission and reflection characteristics of the skin, the omnidirectional IR intensity distributions of 3−5 μm and 8−14 μm at different Mach numbers are obtained. In addition, the exhaust plume IR characteristic for different waves and wavebands are investigated by considering the presence or absence of base and the difference in nozzle inlet temperature. On this basis, the integrated interaction between skin and exhaust plume and their IR characteristics are investigated. Based on the results, the following conclusions can be drawn as follows:

(1) The integral radiation intensity distribution of aircraft skin depends on the Mach number. The radiation intensity emitted by the skin is concentrated in the 8−14 μm waveband. In addition, the upper surface mainly reflects the solar radiation in the 3−5 μm waveband, and the lower surface mainly reflects the ground radiation in the 8−14 μm waveband. To reduce the IR signal of skin, it is necessary to reduce the visible area and reduce the emissivity and reflectance of skin material.
(2) The radiation intensity of the exhaust plume is mainly concentrated in the 3−5 μm waveband. The radiation from the nozzle base has a great influence on the radiation intensity distribution of the exhaust plume, and the backward radiation intensity increases significantly when the effect of the nozzle base is considered.
(3) The aircraft IR characteristic distributions of 3−5 μm waveband are concentrated in the exhaust plume and the high-temperature skin near the exhaust plume, while the IR signals of 8−14 μm waveband are concentrated in the skin. To reduce the IR signal of the nozzle, it is better to reduce the temperature of the core area of the exhaust plume and adopt low-emissivity materials in these parts.

## Data Availability

Not applicable.

## Nomenclature

*A*_*i*_	area of surface element i
**B**	derivative of shape function
*c*_*p*_	heat capacity
$\bar{d}$	spacing of the spectral line
**D**	heat conduction coefficient matrix
*E*_dir_	solar direct radiation
*E*_ref_	secondary radiation from the ground
*E*_sca_	solar scattering radiation
**f** ^e^	loading matrix of element
**g**	temperature gradient matrix
*h*	convective heat transfer coefficient
*H*_sun_	solar constant
*H*_grd_	heat flux emitted from the ground
$I_{b\lambda }(s)$	spectral radiation intensity of blackbody at position s
$I_{\lambda }(s,s)$	spectral radiation intensity along the **s** direction at position s
$I_{b\lambda ,i}$	blackbody spectral radiation intensity of the i-th grid with temperature T_i_
*k*	thermal conductivity
**K** ^e^	stiffness matrix of element
*m*	atmosphere mass
*M*	amount of surface element j that reflects solar radiative energy to surface element i
*Ma*_∞_	Mach number at infinity
*n*_air_	coefficient of temperature dependence
*N*	shape function of a certain node
*p*	atmospheric transparency
*P*	total pressure
*P*_*a*_	gas partial pressure
Pr	Prandtl number
*q*_wb_	heat flux of hemispheric surface for blunt body
*q*_wc_	heat flux of the blunt cone body with a non-stagnation region
*q*_ws_	heat flux of stagnation region
*q* ^*^	specified heat flux
*r*_air_	air-broadened half-width
*r*_self_	self-broadened half-width
$R_{i}^{\det}$	shielding factor of surface element i for this detection angle
*R*_*N*_	radius of curvature of the wall in a certain point N
Re	Reynolds number
*S*_*i*_	integrated line intensity of the i-th spectral line
*t*	temperature of a certain node
*T*_0_	initial temperature
*T*_e_	outer edge temperature of the boundary layer
*T*_w_	outer edge temperature of the wall
*T*_∞_	fluid temperature
*u*_*e*_	velocity for the outer boundary layer
*u*_∞_	incoming flow velocity
*u* ^*^	velocity in the reference enthalpy state
*X*_*j*,*i*_	angle coefficient of surface element i and surface element j
**Greek symbols**	
$\alpha_{i}^{n}$	absorptivity of surface element i in the n-th spectral band
*γ*	specific heat of gas
*γ*_∞_	incoming air specific heat ratio at infinity
$\bar{\gamma }$	average half-width of the spectral line
$\varepsilon_{i}^{n}$	emissivity of surface element i in the n-th spectral band
*η*	wavenumber
*η*_0*i*_	central wave number of the i-th spectral line
*θ*	solar incident angle
*θ*_*i*_	angle of detector normal and plane element normal
$\kappa_{\eta }^{i}$	absorption coefficient contribution of i-th spectral line at η within truncated wavenumber
$\bar{\kappa }$	absorption coefficient of the spectral line
$\kappa_{\lambda }(s)$	absorption coefficient of the gas at position s
*ρ*_e_	density for the outer boundary layer
*ρ*_grd_	average reflection coefficient of the ground.
*ρ*_∞_	incoming flow density
*ρ* ^*^	density in the reference enthalpy state
τ	solar elevation angle
$\tau_{\lambda ,j}$	spectral optical thickness of the j-th grid
𝜑	the angle between the plane of the micro-element and the horizontal plane
ω	central angle based on the body axis
Ω_*c*_	contribution of heat conduction
Ω_*h*_	contribution of convection
Ω_*q*_	contribution of external heat flux
